# Effect of Warming and Elevated O_3_ Concentration on CO_2_ Emissions in a Wheat-Soybean Rotation Cropland

**DOI:** 10.3390/ijerph16101755

**Published:** 2019-05-17

**Authors:** Yuanyuan Wang, Zhenghua Hu, A. R. M. Towfiqul Islam, Shutao Chen, Dongyao Shang, Ying Xue

**Affiliations:** 1 Collaborative Innovation Center on Forecast and Evaluation of Meteorological Disasters, School of Applied Meteorology, Nanjing University of Information Science & Technology, Nanjing 210044, China; wangyuanyuan855@163.com (Y.W.); chenstyf@aliyun.com (S.C.); 20171203270@nuist.edu.cn (D.S.); Pamtale2741176@gmail.com (Y.X.); 2 Department of Disaster Management, Disaster Management E-Learning Centre, Begum Rokeya University, Rangpur 5400, Bangladesh

**Keywords:** global warming, elevated O_3_, CO_2_ emission, winter wheat, soybean

## Abstract

A deeper understanding of the effects of experimental warming and elevated ozone (O_3_) concentration on carbon dioxide (CO_2_) fluxes is imperative for reducing potential CO_2_ emissions in agroecosystems, but are less understood particularly in rotational wheat (*Triticum aestivum*)—soybean (*Glycine max*) croplands. In order to understand such effects on CO_2_ fluxes from winter wheat-soybean rotation, a field experiment was conducted by using the open-top chamber (OTCs) during the growing seasons of 2012 and 2013 at an agro-ecological station in southeast China. The experimental treatments included the control (CK), experimental warming (T, crop canopy temperature increased by ~2 °C), elevated O_3_ concentration (O, O_3_ concentration about 100 ppb) along with temperature enhancement (OT, elevated ~2 °C temperature plus 100 ppb O_3_). The results showed that warming significantly increased the mean CO_2_ fluxes (*MCF*) and the cumulative amount of CO_2_ (*CAC*) from soil and soil-crop systems, while elevated O_3_ and warming enhancement (OT) significantly reduced *MCF* and *CAC*. Besides, warming significantly reduced the biomass of winter-wheat, but it insignificantly decreased the biomass of soybean in the harvest period. The O and OT treatments significantly reduced the biomass of winter-wheat and soybean cropping systems in the harvest time. Both warming and elevated O_3_ concentration decreased the temperature sensitivity coefficients (*Q*_10_) in soil respiration during the experimental period. Overall, our results indicate that elevated O_3_ concentration compensates the effect of warming on CO_2_ emission to some extents, which has a positive feedback impact on the climate system.

## 1. Introduction

Global warming and surface ozone (O_3_) pollution are important global environmental issues nowadays. The global surface-temperature is expected to increase by 1.8~4.0 °C on average by the end of the 21st century due to the increasing concentrations of atmospheric greenhouse gases [1]. Carbon dioxide (CO_2_) is the most important greenhouse gas, and its concentration reached 406 ppm in 2017, exceeding the preindustrial age level by about 45.5% [2]. Similarly, ozone (O_3_) is a highly reactive and anthropogenic greenhouse gas. Tropospheric O_3_ levels are very elevated in Asia [3] and continue to rise [4]. For example, the tropospheric O_3_ concentration has increased at a rate of 0.3~2% in the Northern Hemisphere due to rapid population growth and the use of fossil fuels in the past century [5]. The global tropospheric ozone concentration is anticipated to increase by 20~25% by 2050 [6] and 40~60% by 2100 [7]. Southeast China, one of the most important rotational wheat-soybean producing regions, is under the serious threat of elevated O_3_ levels [8], thus, the effect of global warming and elevated O_3_ concentration on CO_2_ emission in agroecosystems is a global concern to scientists, governments, and the general public [9]. Several O_3_ exposure experiments have used closed chambers, open-top chambers, or free-air O_3_ enrichment systems to explain the responses of different ecosystems to elevated O_3_ [3,8,10].

For instance, agroecosystems are considered to be an important source and sink of CO_2_ which accounts for 10~12% of global anthropogenic CO_2_ emissions [11]. Soil respiration usually refers to the total CO_2_ effluxes at the soil surface, including microbial respiration, root respiration, fauna respiration and chemical oxidation of carbonaceous minerals in soil [12], which are the main output pathways of CO_2_ exchange between the soil and the atmosphere in the terrestrial ecosystem. Hence, even small changes in the size of soil CO_2_ fluxes can have a large impact on atmospheric CO_2_ concentrations [13] and constitute a powerful positive feedback to the climate system.

Temperature is a critical environmental factor in regulating soil C cycling [14], which can directly affect soil respiration by altering the activities of plant roots and soil microbes and can indirectly affect soil respiration by changing plant growth and substrate supply [15,16]. Elevated O_3_ concentrations are reported to have a considerable impact on agricultural cropland in Europe [17] and Asia [18]. Agroecosystems are highly sensitive to elevated O_3_ levels, which cause tremendous crop yield losses [19]. Additionally, elevated O_3_ concentrations are known to reduce C allocation to roots [20] and to alter soil C fluxes by altering rhizosphere deposition and root turnover [17,21]. Elevated O_3_ concentrations may alter soil biological processes by changing the soil physical conditions and regulating the availability of microbial C substrates, which may indirectly affect soil respiration [17], thereby affecting the carbon budget of the whole farmland ecosystem. Furthermore, Andersen reported that elevated O_3_ would affect soil-root respiration and microbial activity by changing plants’ below-ground processes [22]. Several studies have shown that elevated O_3_ affects soil microbial activities [23,24]. Islam et al. hypothesized that the changes of soil-root respiration and soil microbiological procedures may affect the carbon (C) cycle in soils [18], thus influencing carbon dioxide (CO_2_) emissions from plant-soil croplands [25].

Although the individual effects of warming or elevated O_3_ concentration on soil CO_2_ emissions have been investigated and extensively analyzed in different ecosystems, there is a knowledge gap about the combined effects of warming and elevated O_3_ interactions [8]. The results concerning the impacts of warming and elevated O_3_ concentration on CO_2_ emissions are quite contradictory. Warming was reported to increase CO_2_ emissions [26,27], while other studies have found that warming decreases them [28,29] or has no effect on CO_2_ emissions [16,30]. Also, many scholars have hypothesized that elevated O_3_ concentrations reduce CO_2_ emissions [31,32,33,34], while others found the opposite [35,36,37] or no change [17,38]. In addition, the combined effects of simulated warming and elevated O_3_ concentration on CO_2_ emission fluxes in croplands have not been reported. To the best of our knowledge, there is no research on winter-wheat and soybean croplands’ response to the effects of warming and elevated O_3_ concentration on CO_2_ emission fluxes to date.

We hypothesized that soil respiration might be greatly affected by the combined impacts of stimulating warming and elevated O_3_ concentration. To test this hypothesis, we investigated the responses of CO_2_ emission in the winter-wheat and soybean rotation croplands under warming and elevated O_3_ concentration conditions, both individually and combinedly, with open-top chambers (OTCs). The relationship between crop biomass in the harvest time and CO_2_ fluxes with soil temperature was also studied. The specific aims were to examine: (1) if warming and elevated O_3_ concentration would have a large impact on CO_2_ fluxes; (2) if warming and elevated O_3_ concentration would greatly influence crop biomass in the harvest (3) whether CO_2_ fluxes had an evident relationship with soil temperature.

## 2. Materials and Methods

### 2.1. Site Description

The field experiment was set up on cropland at the Agricultural and Ecological Experiment Station (32°03′N, 118°51′E), Nanjing University of Information Sciences and Technology, Jiangsu Province, in south-east China. Winter wheat (*Triticum aestivum*)—soybean (*Glycine max*) is the main crop rotation production regime in the area. The annual average temperature of the experimental site is 15.6 °C, and the annual rainfall averages about 1100 mm. The soil (0 to 20 cm depth) was classified as hydromorphic, the pH (H_2_O) was 6.24, the total organic C and N contents were 19.4 and 1.45 g·kg^−1^, respectively.

### 2.2. Experimental Design

The field experiment was carried out during the winter wheat (between November 2012 and May 2013) and soybean (between June 2013 and November 2013) growing seasons. The major growth stages and fertilization schedules of the winter wheat (cv. Ningmai 13) and soybean (cv. Bayuedou) growing seasons are listed in Table 1.

We set up four treatments: the control (CK), experimental warming (T, crop canopy temperature increased by about 2 °C), elevated O_3_ concentration (O, O_3_ concentration in air was about 100 ppb), and a combination of T and O treatments (OT, elevated about 2 °C temperature plus 100 ppb O_3_), each treatment had three replicates. All treatments were performed in the open-top chambers (OTCs). OTC (2.5 m high and 3.0 m in diameter) was composed of steel frames and covered with clear plexiglass, which can reduce solar radiation by 11%. Totally, there were 12 OTCs in the field.

Warming and O_3_ fumigation began from the seedling stage to maturity stage in the crop growing season. Plants were fumigated with elevated O_3_ concentration during 08:00–18:00 every day (except on rainy days).

An infrared radiator consisting of three infrared radiation lamps (500 W, 220 V, 120 cm length) was hung about 1 m above the crop canopy for 24-hour continuous heating of each warmed OTC (T and OT treatments). In the unheated OTCs (CK and O treatments), dummy lamps of the same configuration were suspended. Throughout the experimental period, the temperature was continuously recorded, and the crop canopy temperature in warmed OTCs was increased by approximately 2 °C.

O_3_ generators (Wohuan Inc., Nanjing, China) was used for O_3_ fumigation in the respective OTCs. The O_3_ concentration was monitored by O_3_ analyzers (Aeroqual Inc., Auckland, New Zealand) during the crop growth period. Solenoid valves and electromagnetic valves, connected to a programmable Log Controller (Wohuan Inc.), were used to control gas meters to furnish the specified O_3_ concentration. Diurnal variation of O_3_ concentration of each treatment is shown in Figure S1. Ozone concentrations in O and OT treatments are kept around 100 ppb, from 08:00 to 18:00, during the winter wheat and soybean growing seasons (Figure S1). Under the ozone fumigation, the monitored average O_3_ concentration (from 08:00 to 18:00) of wheat and soybean experiments were 102.05 ppb and 100.35 ppb, respectively. Meanwhile, no ozone fumigation treatment, the average O_3_ concentration (from 08:00 to 18:00) of wheat and soybean experiments were 79.08 ppb and 66.60 ppb, respectively.

### 2.3. CO_2_ Flux Measurement

The CO_2_ emission fluxes were measured by a static chamber-gas chromatograph technique [39]. During the gas sampling periods, boardwalks were installed to reduce soil and crop disturbance. Two circular base frames (8 cm high and 22 cm in diameter) for the sampling chamber were installed in each OTC, one base frame contained crops to observe CO_2_ flux of the soil-crop system, and the plants were removed from the other base frame to measure CO_2_ flux in the soil. There was a 2.5 cm wide groove on the top rim of each base frame. The sampling chamber was a 100 cm high polyvinyl chloride (PVC) cylinder. In order to minimize the impact of solar radiation on the internal temperature, each sampling chamber was wrapped in one layer of sponge and aluminum foil. Water was filled into the groove on the top rim of the base frame to seal the chamber during the sampling. The syringes were used to collect gas samples once or twice a week at 0, 10, and 20 min after the gas chamber was closed. Gas samples were taken between 9:00 and 11:00 a.m. Beijing time (GMT+8). The sampling time is based on the diurnal variation model of gas emissions, assuming that the model remains unchanged for one day [39].

The mixing ratios of CO_2_ were analyzed by a modified gas chromatograph (Agilent 4890D, Agilent Co., Santa Clara, CA, USA) equipped with an Electron Capture Detector (ECD) [40]. The slope of the mixing ratio changing with values at 0, 10, and 20 min after the chamber sealing was used to calculate CO_2_ fluxes [39]. Almost all the samples reached the linear regression values of *R*^2^ > 0.90, which indicated that the measurement had good accuracy. The CO_2_ emission flux, i.e. the variation of CO_2_ emission per unit area per hour in soil, is calculated using the following formula:

(1) $$F=\frac{\Delta m}{A\cdot \Delta t}=\frac{\rho \cdot V\cdot \Delta C}{A\cdot \Delta t}=\rho \cdot H\cdot \frac{\Delta C}{\Delta t}\;,$$

where *F* is the CO_2_ emission flux (mg m^−2^·h^−1^); *ρ* is CO_2_ density under standard conditions (1.96 kg·m^−3^); ΔC and Δm are the mixed CO_2_ concentration (ppm) and the gas mass (mg) in the chamber during a given period (Δt), respectively; *H*, *V* and *A* are the height (m), volume (m^3^) and bottom area (m^2^) of the chamber, respectively.

The seasonal cumulative amount of CO_2_ (*CAC*) emission was sequentially accumulated from the emissions between each pair of adjacent intervals of the measurement. The concrete formulas are as follows:

(2) $$CAC=\sum_{i=1}^{n}(\frac{F_{i}+F_{i+1}}{2})\cdot \left(t_{i+1}-t_{i}\right)\cdot 24\;,$$

where CAC is the cumulative amount of CO_2_ (mg·m^−2^), *F* is the CO_2_ flux (mg m^−2^·h^−1^), *i* is the first sampling, *t*_*i*+1_ − *t_i_* is the interval between two determination dates (d), *n* is the total number of measurements.

### 2.4. Temperature and Soil Moisture Measurement

Air temperature in the sampling chamber was recorded synchronously with each set of CO_2_ emission measurements during the sampling period. A soil temperature-moisture instrument (TZS-IW, Tuopu Ltd. Hangzhou, China) was used to measure soil temperature and soil moisture at a 5-cm depth at a location close to the base frame.

### 2.5. Crop Biomass Measurements

A drying oven was used to determine the biomass of the crop, each biomass sample was inactivated after drying at 105 °C for 1 h and then dried at 80 °C for more than 48 h. 

### 2.6. Data Analysis

The following exponential function was employed to describe the relationship between soil respiration rate and temperature:

(3) $$R_{S}=ae^{bT},$$

where *R_S_* is the soil respiration rate, *T* is soil temperature at 5 cm, the coefficient *a* is the intercept of *R_S_* at 0 °C, and coefficient b represents the temperature sensitivity of *R_S_*, respectively. The value of *Q*_10_ (the increasing multiples of the soil respiration rate when the temperature increases by 10 °C) was then calculated as:

(4) $$Q_{10}=e^{10b},$$

The average CO_2_ fluxes and their errors were calculated from three replicates. ANOVA test was applied to evaluate the effects of warming and elevated O_3_ concentration on the mean CO_2_ flux, the cumulative amount of CO_2_ and biomass among the four treatments. The significance level is at the *p* = 0.05 in this study. SPSS version 19.0 (SPSS Inc., Chicago, IL, USA) was used to perform all statistical analyses.

## 3. Results

### 3.1. Changes of Soil Temperature and Moisture

**Figure 1:**
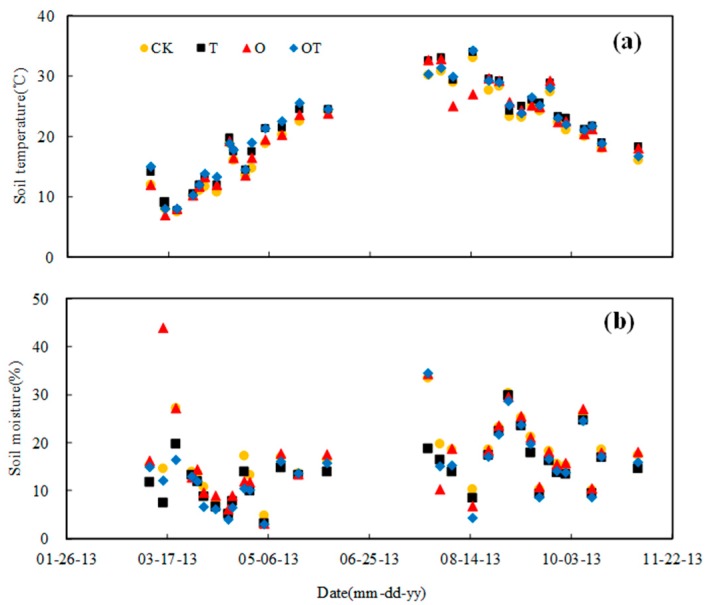
Seasonal variation in soil temperature and moisture: (**a**) Temperature in the winter wheat and soybean-growing seasons; (**b**) Moisture in the winter wheat and soybean-growing seasons.

The seasonal variations of soil temperature and moisture at 5 cm depth during the winter-wheat and soybean growing seasons are shown in Figure 1. Soil temperature showed seasonal trend patterns following the seasonal variation in air temperature, exhibiting a rising trend during the winter wheat growth stages, and a decline in the soybean growth stages (Figure 1a). During the study period, there were no significant differences in soil temperature among different treatments (*p* > 0.05). Soil temperature ranged from 7–35 °C. Compared with the CK treatment, the mean soil temperature of T, O, and OT treatments increased 1.3, 0.46, and 1.6 °C in the winter-wheat growing season, and increased 1.32, 0.07, and 1.02 °C in the soybean growing season, respectively. There was no clear seasonal change in soil moisture, and no differences in soil moisture among different treatments, for the sampling dates (Figure 1b). Soil moisture was between 3.17 and 43.9%. Compared with the CK treatment, the average soil moisture content of T and OT treatments decreased by 2.6% and 2.7% during the experimental period, respectively.

### 3.2. Seasonal Change of CO_2_ Emissions in Soil-Crop System

**Figure 2:**
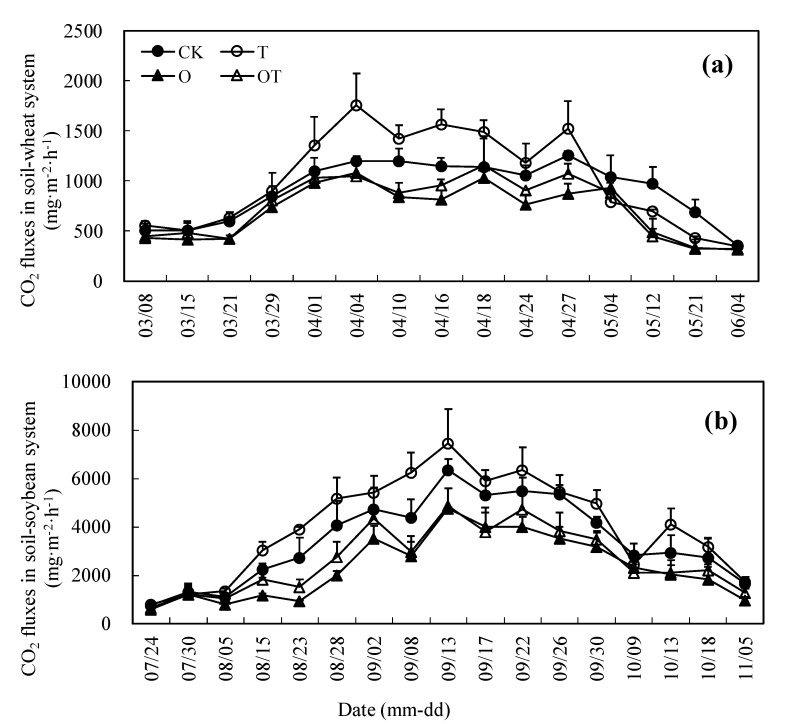
Effect of warming and elevated O_3_ concentration on CO_2_ emission fluxes from soil-crop system: (**a**) CO_2_ emission fluxes from soil-winter wheat system; (**b**) CO_2_ emission fluxes from soil-soybean system. Data are the mean values. Error bars are SEs. In figure (a), 03-08 to 03-28 are the turning-green stage, 03-29 to 04-09 are the elongation-booting stage, 04-10 to 04-26 are the heading-flowering stage, and 04-27 to 06-04 are the grain filling-maturity stage. In figure (b), 07-24 to 08-14 are the branching stage, 08-15 to 09-21 are the flowering-pod stage, and 09-22 to 11-05 are the grain filling-maturity stage.

CO_2_ emissions in the soil-wheat system displayed similar patterns for different treatments (Figure 2a). In general, the seasonal trend of CO_2_ emission from all treatments showed an increasing trend and then a decline. The CO_2_ fluxes were lower at the turning-green stage, showed a rising trend during the elongation-booting stage and were steady at the heading-flowering stage, then gradually slowed down during the late stage with crop growth. Figure 2b shows a similar pattern of CO_2_ emissions in the soil-soybean system for different treatments. The seasonal trend of CO_2_ emission from all treatments exhibited an increasing trend and then a decline. The CO_2_ fluxes demonstrated a rising trend during the branching and flowering-pod stages, then gradually slowed down during the late crop growth stage. It is noteworthy that warming and elevated O_3_ concentration did not alter the seasonal patterns of CO_2_ emission from the soil-wheat and soybean cropping systems (Figure 2).

### 3.3. Effects of Warming and Elevated O_3_ Concentration on the Mean CO_2_ Emission from Soil-Crop System

The mean CO_2_ fluxes (MCF) from the soil-crop system during the winter-wheat and soybean croplands are presented in Table 2. In the winter wheat-growing season, compared with the CK treatment, the O and OT treatments significantly reduced the MCF in the turning-green stage. In the elongation-booting stage, T treatment significantly increased the MCF. In the heading-flowering stage, T significantly increased the MCF, whereas O decreased it. In the grain filling-maturity stage, T, O, and OT treatments all significantly reduced the MCF. According to the whole growth stage, the MCF in different treatments followed the sequence: T > CK > OT > O. In comparison with CK, T treatment significantly enhanced the MCF, whereas O and OT treatments significantly reduced it.

In the soybean growing season, compared with the CK treatment, T treatment significantly enhanced the *MCF*, while O and OT significantly reduced it, in the flowering-pod and grain filling-maturity stages. According to the whole growth stage, the *MCF* in different treatments was in the order of T > CK > OT > O. In comparison with CK, T treatment significantly increased the *MCF*, while O and OT treatments significantly decreased it.

### 3.4. Seasonal Change of CO_2_ Emissions from Soil

**Figure 3:**
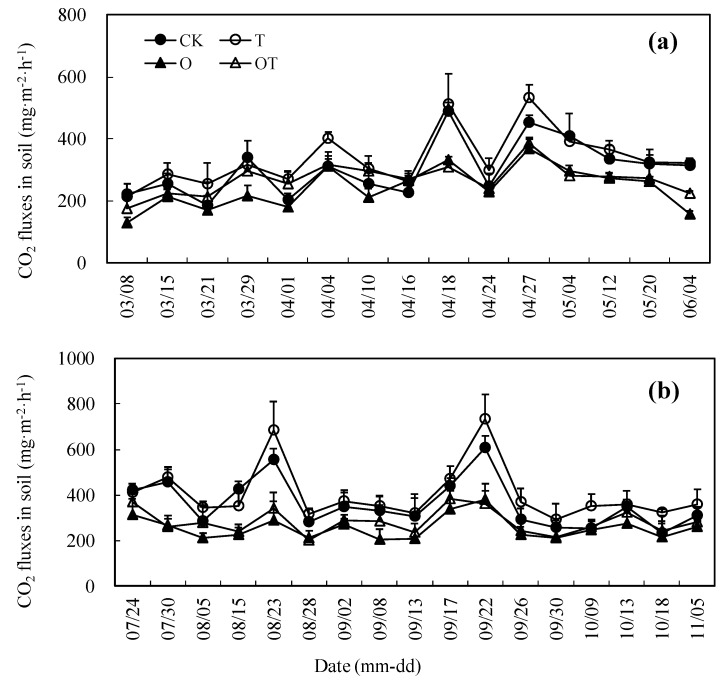
Effect of warming and elevated O_3_ concentration on CO_2_ emission fluxes from soil: (**a**), (**b**) Soil CO_2_ emission fluxes in winter wheat and soybean-growing seasons, respectively. Data are the mean values. Error bars are SEs. In figure (**a**), date 03-08 to 03-28 are the turning-green stage, 03-29 to 04-09 are the elongation-booting stage, 04-10 to 4-26 are the heading-flowering stage, and 04-27 to 06-04 are the grain filling-maturity stage of winter wheat. In figure (**b**), date 07-24 to 08-14 are the branching stage, 08-15 to 09-21 are the flowering-pod stage, and 09-22 to 11-05 are the grain filling-maturity stage of soybean.

CO_2_ emissions from soil in winter-wheat farmland followed similar trends for different treatments (Figure 3a). In general, the seasonal trend of CO_2_ emission from all treatments demonstrated an increasing trend and then decrease. The CO_2_ fluxes exhibited an upward trend before the grain filling stage, then gradually slowed down at the late stage with crop growth. Figure 3b demonstrates a similar pattern of CO_2_ emissions from the soil in soybean farmland for different treatments. The seasonal trend of CO_2_ emission from all treatments exhibited a rotational trend and turns, ultimately declining. It is observed that warming and elevated O_3_ concentration did not alter the seasonal patterns of CO_2_ emission from the soil in the winter-wheat and soybean growing seasons (Figure 3).

### 3.5. Effects of Warming and Elevated O_3_ Concentration on the Mean CO_2_ Fluxes from Soil

The *MCF* values from the soil in the winter-wheat and soybean growing seasons are displayed in Table 3. In the winter-wheat growing season, compared with the CK treatment, O treatment significantly decreased the *MCF* in the turning green stage. In the elongation-booting stage, T treatment significantly increased the *MCF*, whereas O treatment reduced it. In the heading-flowering stage, T treatment significantly enhanced the *MCF*. In the grain filling-maturity stage, T treatment significantly increased the *MCF*, whereas O and OT treatments significantly decreased it. According to the whole growth stage, the *MCF* from the soil in different treatments followed the order of T > CK > OT > O. In comparison with CK, T treatment significantly enhanced the *MCF*, while O and OT treatments significantly reduced it.

In the soybean-growing season, compared with CK, O treatment significantly decreased the *MCF* in the branching stage. In the flowering-pod stage, O and OT treatments significantly decreased the *MCF*. In the grain filling-maturity, O treatment significantly reduced the *MCF*. According to the whole growth stage, the *MCF* from the soil in different treatments was T > CK > OT > O. In comparison with CK, O treatment significantly reduced the *MCF*, while O and OT treatments change it slightly.

### 3.6. Effect of Warming and Elevated O_3_ Concentration on Cumulative Amount of CO_2_ Emission from Soil and Soil-Crop System

**Figure 4:**
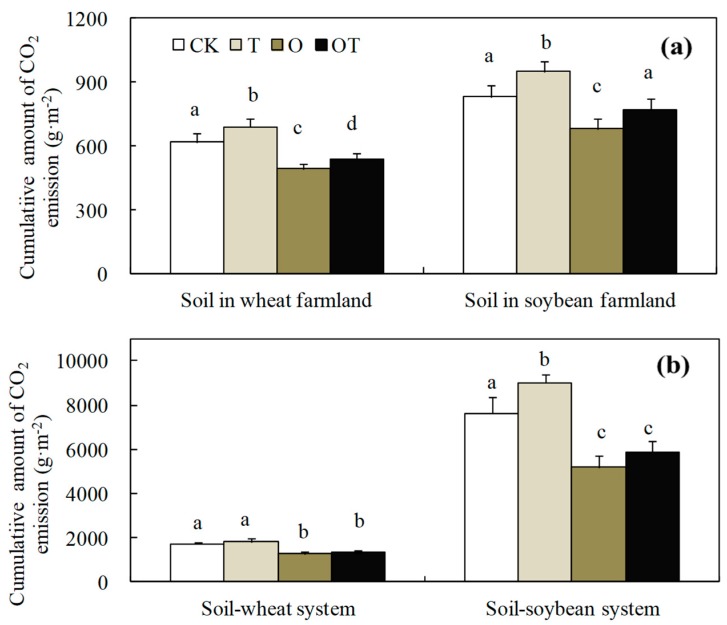
Effect of warming and elevated O_3_ concentration on cumulative amount of CO_2_ emission from soil and soil-crop system. Figure (**a**) and (**b**), cumulative amount of CO_2_ emission from soil and soil-crop system, respectively. Data are the mean values. Error bars are SEs. Different lowercase letters denote significant difference among different treatments at *p* ≤ 0.05.

The cumulative amount of CO_2_ (*CAC*) emissions from the soil and soil-crop system are presented in Figure 4. In the winter wheat growing season, compared with the CK treatment, T treatment enhanced the *CAC* from soil in winter-wheat farmland by 11.8% (*p* < 0.05), whereas O and OT treatments decreased by 20.2% (*p* < 0.05) and 12.7% (*p* < 0.05), respectively. Similarly, O and OT treatments decreased the *CAC* from soil-winter wheat system by 25.6% (*p* < 0.05) and 22.3% (*p* < 0.05). The *CAC* from soil and the soil-winter wheat system in different treatments was in the sequence e.g., T > CK > OT > O.

In the soybean growing season, compared with CK, T treatment increased the *CAC* from soil in soybean farmland by 14.5% (*p* < 0.05), while O treatment decreased by 17.9% (*p* < 0.05). In addition, T treatment enhanced the *CAC* from soil-soybean system by 18.0% (*p* < 0.05), whereas O and OT treatments decreased by 32.1% (*p* < 0.05) and 23.0% (*p* < 0.05), respectively. The *CAC* from soil and soil-soybean systems in different treatments had followed the sequence: T > CK > OT > O.

### 3.7. Effect of Warming and Elevated O_3_ Concentration on Biomass of Winter-Wheat and Soybean

The biomasses of winter-wheat and soybean are shown in Table 4. In the winter wheat-growing season, the total biomass order of the different treatments was T > OT > O > CK during the elongation-booting to the grain filling stages, while in the harvest period it was in the order: CK > T > O > OT. Compared with CK, the T and OT treatment significantly increased the shoot biomass and total biomass in the elongation-booting and heading-flowering stage. In the grain filling stage, compared with CK, T treatment significantly increased the shoot biomass and total biomass, O treatment significantly reduced it. In the harvest period, compared with CK, T, O, and OT treatments all significantly reduced the shoot, root, and total biomass. It is evident that warming significantly increased the biomass of winter wheat in the elongation-booting to the grain filling stages, but reduced it in the harvest. Elevated O_3_ concentration significantly reduced the biomass of winter wheat in the grain filling and the harvest period, but had no significant effect on the biomass in the elongation-booting and heading-flowering stages. The combination of warming and elevated O_3_ concentration significantly increased the biomass of winter wheat in the elongation-booting and heading-flowering stages, but reduced it in the harvest.

In the soybean-growing season, the total biomass in the harvest for different treatments had followed the order of CK > T > O > OT. Compared with the CK treatment, T treatment insignificantly reduced the shoot, root, and total biomasses; O treatment significantly reduced the shoot biomass and total biomass, but it insignificantly reduced the root biomass; OT treatment significantly reduced the shoot biomass and total biomass, while it insignificantly reduced the root biomass. It is obvious that elevated O_3_ concentration and the combination of warming and elevated O_3_ concentration significantly reduced the biomass of soybean, whereas warming insignificantly decreased the biomass of soybean, in the harvest time.

### 3.8. Effect of Warming and Elevated O_3_ Concentration on the Temperature Sensitivity of Soil Respiration

**Figure 5:**
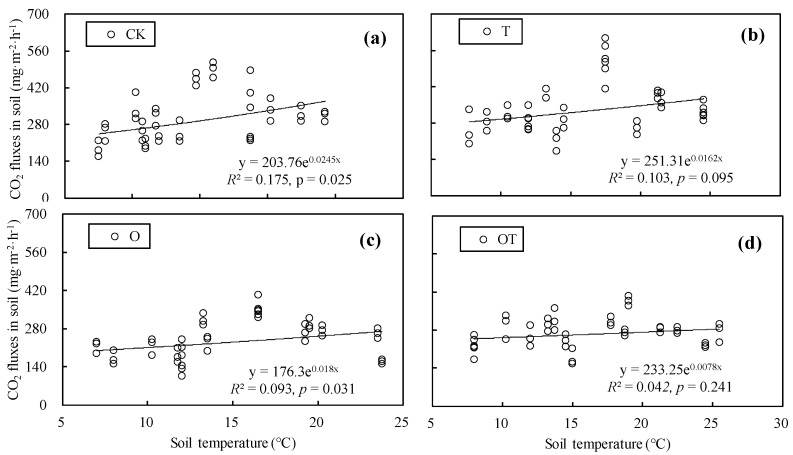
Relationship between soil respiration rates and soil temperature in winter-wheat farmland under different treatments. (**a**), (**b**), (**c**), and (**d**), the relationship between soil respiration rates and soil temperature in winter-wheat farmland under the CK, T, O, and OT treatments, respectively.

**Figure 6:**
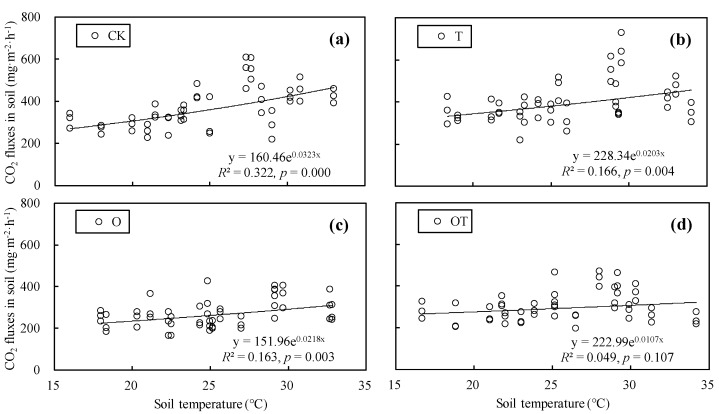
Relationship between soil respiration rates and soil temperature in soybean farmland under different treatments. (**a**), (**b**), (**c**), and (**d**), the relationship between soil respiration rates and soil temperature in soybean farmland under the CK, T, O, and OT treatments, respectively.

We conducted a regression analysis on soil respiration rates and soil temperature in winter-wheat and soybean farmlands for the CK, T, O, and OT treatments, respectively. Our results showed that there was an exponential relationship between soil respiration rates and soil temperature in winter-wheat and soybean systems (Figure 5 and Figure 6). According to the exponential regression equation, the $Q_{10}$ values for soil respiration in winter-wheat farmland for the CK, T, O, and OT treatments were 1.278, 1.176, 1.197, and 1.081, respectively (Figure 5), similarly, the *Q*_10_ values for soil respiration in soybean farmland for the CK, T, O, and OT treatments were 1.381, 1.225, 1.244, and 1.113, respectively (Figure 6). The *Q*_10_ values for soil respiration in winter-wheat and soybean systems in different treatments were all expressed as CK > O > T > OT.

## 4. Discussion

### 4.1. Warming and Elevated O_3_ Concentration Affect CO_2_ Emission Fluxes

#### 4.1.1. Seasonal Change of CO_2_ Emission Fluxes

Soil respiration mainly includes autotrophic and rhizosphere respiration of plant roots and heterotrophic decomposition of soil organic carbon [41]. CO_2_ emissions from the soil-crop system refer to soil surface respiration and plant above-ground respiration. Warming and elevated O_3_ concentration may affect CO_2_ emission by changing crop growth, soil properties, and soil microbial activity [16,17]. Our results showed that warming and elevated O_3_ concentration did not change the seasonal patterns of CO_2_ emission fluxes from the soil and soil-crop systems in the winter wheat and soybean growing seasons. The seasonal change patterns of CO_2_ emissions in the soil during the winter-wheat and soybean growing seasons in the soil and soil-crop systems of each treatment were similar, showing a trend of the first rising and then decreasing with crop growth. In the early growth stage of crops, the crops grown slowly had less aboveground biomass and lower coverage, so the CO_2_ emissions in the soil and soil-crop systems was relatively low. Subsequently, with the crop growth, the shoot and root biomasses of the crops increased gradually, thereby enhanced the CO_2_ emissions in the cropland. However, in the later growth stage of crops, plants had much stronger carbon absorption and utilization, and soil available carbon decreased with time, resulting in lower CO_2_ emission fluxes from the soil and soil-crop systems. The soil CO_2_ emission is mainly affected in a complex way by temperature, moisture, soil properties, root exudation, and the quality and quantity of decomposing organic substrates [42]. It is quite difficult to explain the specific mechanisms in details on account of the fact some processes are intertwined.

#### 4.1.2. Warming Affect CO_2_ Emission Fluxes

Temperature is a critical environmental factor in regulating soil C cycling [14], which can directly affect soil respiration by altering the activities of plant roots and soil microbes and can indirectly affect soil respiration by changing plant growth and substrate supply [15,16].

In most natural ecosystems, warming significantly enhanced CO_2_ emissions [26,27,43,44], for example, a meta-analysis by Rustad et al. reported that on average, warming of 0.3–6 °C significantly increased soil respiration rates by 20% [43]. In addition, Bergner et al. indicated that warming increased soil CO_2_ flux by 20% in a burned boreal forest of Alaska [44]. However, Reth et al. did not observe a significant change in soil respiration after a 10-year long-term warming experiment in a farm of southern Germany [30]. By contrast, Liu et al. showed that warming reduced total soil respiration and microbial respiration in a semiarid grassland of northern China, which could be attributed to warming-induced water stress, and inhibited plant growth [28]. Furthermore, a 10-year soil warming study by Melillo et al. reported that warming promoted the decomposition of soil organic carbon and increased the carbon released into the atmosphere, which only happened in the early stage of warming. In the late stage of warming, the effect of warming on CO_2_ production was insignificant [45]. Similarly, Luo et al. conducted a field warming experiment in the grassland of Oklahoma in the USA, and stated that the warming significantly promoted the soil respiration of the grassland in the early stage of warming. As time went on, the response of soil respiration to warming weakened, resulting in an adaptation of warming [46]. The reason for this adaptation may be due to changes in soil microbial communities and the restriction of soil nutrients on soil respiration.

Our results showed that warming of ~2 °C significantly increased CO_2_ emissions in soil and soil-crop systems. A similar result was found by Cheng et al., who pointed out that warming significantly enhanced soil respiration in a rice-wheat rotation agroecosystem [47]. In this study, however, warming reduced CO_2_ emissions during the grain filling-maturity stage in winter-wheat growing season. Furthermore, we observed that winter-wheat of T and OT treatments matured on May 4th, while that of CK treatment matured on May 20th. Warming accelerated the growth of winter-wheat, thus shortened the grain filling stage and promoted early maturity of winter-wheat, which was not conducive to the accumulation of winter-wheat biomass, thus leading to the reduction of CO_2_ emissions in the grain filling-maturity stage.

#### 4.1.3. Elevated O_3_ Concentration Affect CO_2_ Emission Fluxes

Elevated O_3_ concentration is known to reduce C allocation to roots [20] and to alter soil C fluxes by altering rhizosphere deposition and root turnover [17,21]. Elevated O_3_ concentration may alter soil biological processes by changing soil physical conditions and regulating the availability of microbial C substrates, which may indirectly affect soil CO_2_ emissions [17]. However, our results in the wheat-soil system are in disagreement with the findings of Zhang et al. (2010), where no alteration in soil respiration has been reported [48]. Hu et al. (2011) reported a decrease in soil respiration in wheat agroecosystems, which is similar to our results [8]. The main differences occur in the results might be due to various exposures to O_3_ concentrations on soil respiration in wheat-soil agroecosystems [8].

Previous studies have widely reported negative effects [21,31,32,33,34] of elevated O_3_ concentrations on CO_2_ emissions, but positive [35,36,37] or no effects [17,38] of elevated O_3_ concentration on CO_2_ emission have been observed too. For example, Edwards indicated that elevated O_3_ concentration reduced root exudation of organic compounds from the roots, inhibited the supply of inorganic and organic nutrients to soil microorganisms, and ultimately led to a decrease of microbial metabolism and soil respiration [31]. Similarly, King et al. showed that elevated O_3_ concentration significantly inhibited soil respiration in paper birch and trembling aspen [21]. However, Scagel et al. detected seasonal differences of root and soil respiration of ozone-exposed ponderosa pine, and found that root CO_2_ production and Respiration Quotient (RQ, the ratio between CO_2_ production and O_2_ consumption) increased with increasing O_3_ exposure [36]. Kasurinen et al. also observed that elevated O_3_ concentration increased soil CO_2_ efflux of two silver birch clones, which might be related to O_3_ stress promoting mycorrhizal formation, improved nutrient acquisition by roots and root turnover rate [37]. Tingey et al. reported that elevated O_3_ concentration had no significant effect on soil respiration in ponderosa pine [38]. Furthermore, a 3-year open-top chamber experiment by Kanerva et al. showed that in a meadow ecosystem moderately elevated O_3_ concentrations (40–50 ppb) had no effect on the daily CO_2_ fluxes duriung the first year, but the daily CO_2_ fluxes increased in the second year, and the daily CO_2_ fluxes decreased in the last year, [17].

Kou et al. investigated the effects of elevated O_3_ concentration on CO_2_, CH_4_, and N_2_O emissions in a rice-wheat rotation cropland, and concluded that elevated O_3_ concentration significantly increased the CO_2_ emissions in the soil-rice system, but had no effect on the CO_2_ emissions in root-free soil during the rice growing season. In the wheat growing season, elevated O_3_ concentrations significantly increased the CO_2_ emissions in the soil-rice system and root-free soil [3]. Our study shows that elevated O_3_ concentration significantly reduced CO_2_ emissions in the soil and the soil-crop systems, which is consistent with a recent study by Chen et al., who found that elevated O_3_ concentrations inhibit soil respiration in the rotational winter wheat-soybean cropland [34]. Based on the aforementioned discussion, it is clear that disagreement among several studies infers that further studies should be performed on soil respiration in each ecosystem.

#### 4.1.4. The Combination of Warming and Elevated O_3_ Concentration Affect CO_2_ Emission Fluxes

As mentioned above, in our study, warming significantly promoted CO_2_ emissions in the winter wheat-soybean rotation cropland. Related studies have shown that elevated CO_2_ concentrations can mitigate the negative effects of elevated O_3_ concentration on photosynthesis and growth of plants [8,49]. King et al. proved that elevated CO_2_ concentration enhanced the fine root biomass of *Betula papyrifera* and *Populus tremuloides*, and elevated O_3_ concentration had no significant effect on the fine root biomass, while the fine root biomass of the combination of elevated CO_2_ and O_3_ treatments was between that of elevated CO_2_ treatment and that of elevated O_3_ concentration treatment [21]. Thus, warming may alleviate and compensate for the damage to crops caused by elevated O_3_ concentration to a certain extent, thereby the CO_2_ emissions in the croplands might be increased. Our results indicate that the combination of warming and elevated O_3_ concentration treatments significantly reduced CO_2_ emission in the soil and soil-crop systems. The *MCF* and *CAC* from soil and soil-winter wheat systems in different treatments were T > CK > OT > O. It is clear that elevated O_3_ concentration compensates the effects of warming on CO_2_ emission in some extents, and the negative effect of elevated O_3_ concentration was stronger than the positive effect of warming on CO_2_ emission, which positively feedbacks to the current climate change scenarios.

### 4.2. Warming and Elevated O_3_ Concentration Affect Crop Biomass

High temperature may reduce crop photosynthesis [50] and inhibit crop growth [51]. In contrast, warming may increase plant biomass by increasing the metabolism and photosynthesis of plants [45]. Wu et al. indicated that warming increased shoot biomass, but had no significant influence on total biomass and root biomass, and only under continuous warming conditions the total biomass would be significantly increased [52]. Chen et al. showed that warming had no significant effect on the root and shoot biomasses of the rotational winter wheat and soybean croplands [53]. Wada et al. reported that the response of biomass to simulated warming was significantly different among different species [54]. Our results suggest that warming significantly reduces the biomass of winter wheat, but it insignificantly decreases the biomass of soybean during the harvest period. The results also reveal that warming shortened the grain filling stage of winter wheat, which was not conducive to the accumulation of biomass, so the winter-wheat biomass of the warming treatment at the time of the harvest was significantly lower than that of the CK treatment. Walther et al. showed that warming advanced the phenological period of crops in spring, but delayed the phenological period of crops in autumn [55]. The effect of warming on the phenological period of crops might vary with different species and duration of warming.

Elevated O_3_ concentration is known to decrease net plant photosynthesis [56] and dry matter production [57], thus inhibiting plant growth and crop biomass [58]. In fact, Andersen found that elevated O_3_ concentrations insignificantly decreased the root biomass and root-shoot ratio [23]. Kou et al. showed that elevated O_3_ concentration insignificantly reduced the total biomass of wheat but significantly decreased that of rice, and significantly reduced the root-total ratio of wheat but insignificantly increased that of rice [3]. Chen et al. reported that elevated O_3_ concentration significantly reduced soybean biomass, but had no significant effect on winter wheat biomass [34]. Our results indicate that elevated O_3_ concentration treatment and the combination of warming and elevated O_3_ concentration treatments significantly reduced the biomass of winter wheat and soybean in the harvest time. It is observed that winter wheat of O and OT treatments matured on May 4^th^, while that of CK treatment matured on May 20^th^ in winter-wheat growing season. Elevated O_3_ concentration treatment and the combined warming and elevated O_3_ concentration treatments shortened the grain filling stage of winter wheat, which was not conducive to the accumulation of winter wheat biomass, so the winter wheat biomass of elevated O_3_ concentration treatment and the combination of warming and elevated O_3_ concentration treatments in the harvest was significantly lower than that of the CK treatment.

In this study, the root biomass of winter wheat was significantly reduced at harvest, but the effects of warming and elevated concentration on root biomass of winter wheat in the elongation-booting, heading-flowering, and grain filling stages, respectively, were insignificant. The biomass of both winter-wheat and soybean croplands for different treatments in harvest was in the sequence of CK > T > O > OT. It is obvious that warming or elevated O_3_ concentration alone can all inhibits crop biomass in the harvest to some extents, thus the combined warming and elevated O_3_ concentration treatments had the strongest inhibitory effect on crop biomass in the harvest time. In addition, the inhibition of warming on crop biomass in the harvest was weaker than that of elevated O_3_ concentration.

### 4.3. Warming and Elevated O_3_ Concentration Affect the Temperature Sensitivity of Soil Respiration

Soil temperature is a key factor affecting soil respiration, which is mostly explained by the variation of daily changes and seasonal changes in soil respiration [59]. At present, the equations describing the relationship between soil respiration and soil temperature are mainly linear, exponential and Arrhenius equations [30,60,61]. These equations have their own advantages in modeling soil respiration. For example, Lloyd et al. pointed out that the Arrhenius equation has higher activation energy at low temperatures [62], However, it may underestimate the response of soil respiration at low temperature [61]. Compared with the linear equation, the exponential equation can make a better correlation between soil respiration and soil temperature [61], and can calculate the temperature sensitivity coefficients (*Q*_10_) for soil respiration. In this study, if a linear equation was used to describe the relationship between soil respiration and soil temperature, the *R*^2^ for CK, T, O, and OT treatments in winter wheat growing season were 0.137, 0.083, 0.097, and 0.036, and in the soybean growing season they were 0.318, 0.159, 0.165, and 0.052, respectively. Obviously, in general, the use of the exponential equation can make soil respiration and soil temperature more correlate better than linear equations (Figure 5 and Figure 6).

It is often assumed that warming will decrease the temperature sensitivity of soil respiration [42,46]. Luo et al. suggested that warming of 2 °C decreased the value of *Q*_10_, which could result from a reduction in plant production leading to less root respiration, soil drying reducing root and microbial activity, and substrate limitation [46]. However, warming also had no significant effect on the *Q*_10_ value for soil respiration in a rice-winter wheat rotation agroecosystem, in southeast China [47]. In addition, either positive [8] or negative [38] effects of elevated O_3_ concentration on *Q*_10_ values have been reported. Hu et al. pointed out that elevated O_3_ concentrations increased the *Q*_10_ for soil respiration in the soil-winter wheat system, which means that elevated O_3_ concentrations may increase the respiration rate of the ecosystem in the future under a temperature rise scenario [8]. However, a multi-year study carried out by Tingey et al. has found that elevated O_3_ concentration significantly reduced the value of *Q*_10_ in ponderosa pine [38].

Our results showed that there was an exponential relationship between soil respiration rates and soil temperature in winter-wheat and soybean systems. A similar finding was reported by Christian et al., who showed that the exponential equation can better model the relationship between soil respiration and soil temperature, and soil temperature can explain most of the variation in soil respiration [60]. In this study, warming and elevated O_3_ concentration reduced the temperature sensitivity of soil respiration to a certain extent, thus the combination of warming and elevated O_3_ concentration treatments had the strongest inhibitory effect on the value of *Q*_10_. Furthermore, the inhibition of warming on the value of *Q*_10_ was stronger than that of elevated O_3_ concentration. These inhibitions in the *Q*_10_ for soil respiration under warming and elevated O_3_ concentration result from several mechanisms, including less root respiration caused by the reduction of plant yield, the reduction in root and microbial activity due to soil drying, and substrate limitation [38,46]. Under the scenario of simultaneous rise of temperature and O_3_ concentration in the future, the combined effects of warming and elevated O_3_ concentration on the ecosystem respiration rates are complex and variable, however, there are uncertain of the exact mechanism of such effects, which may require further investigation.

## 5. Conclusions

Our experimental results demonstrated that warming and elevated O_3_ concentration did not alter the seasonal patterns of CO_2_ emission from the soil and soil-crop systems in the winter wheat and soybean-growing seasons. The results clearly indicated that warming significantly increased the *MCF* and *CAC* from the soil and soil-crop systems, but elevated O_3_ concentration and the combined warming and elevated O_3_ concentration significantly reduced these parameters. Additionally, warming, elevated O_3_ concentration and the combined treatments significantly reduced the biomass of both winter wheat and soybean cropping systems in the harvest period. We also found that there was an exponential relationship between soil respiration rates and soil temperature in the winter-wheat and soybean farmland. Warming and elevated O_3_ concentration reduced the temperature sensitivity of soil respiration. In conclusion, our results indicate that elevated O_3_ concentration compensates the impact of warming on CO_2_ emission to some extent, which has a significant feedback impact on the climate system. Our study provided evidence for changes in soil carbon emissions in cropland under changing climate conditions in the future.

## Figures and Tables

**Table 1:** Main growth stages and fertilization schedules.

Date	Winter-Wheat	Date	Soybean
2012-11-18	Sow, fertilization 40 g/m^2^ (N 26%, P_2_O_5_ 11.5%)	2013-06-29	Sow, fertilization 32 g/m^2^ (N 18%, P_2_O_5_ 46%)
2012-11-20	Seedling	2013-07-04	Seedling
2013-01-05	Fertilization (urea, 19 g/m^2^)	2013-07-14	Trefoil
2013-02-06	Turning green	2013-07-24	Branching
2013-02-26	Fertilization (urea, 19 g/m^2^)	2013-08-15	Flowering
2013-03-21	Elongation	2013-09-08	Pod
2013-03-26	Booting	2013-09-22	Grain filling
2013-04-16	Heading	2013-11-05	Harvest
2013-04-18	Flowering		
2013-04-27	Grain filling		
2013-05-20	Maturity		
2013-06-04	Harvest		

**Table 2:** Effect of warming and elevated O_3_ concentration on mean CO_2_ emission fluxes from the soil-crop system (mg·m^−2^·h^−1^).

Crop	Growth Stages	Treatments	The Mean CO_2_ Emission Fluxes
Wheat	Turning green	CK	535.00 ± 3.43
		T	565.40 ± 25.51 (+5.7%)
		O	423.06 ± 53.70 (−20.9%) **
		OT	453.46 ± 10.13 (−15.2%) **
	Elongation-Booting	CK	1050.57 ± 64.46
		T	1339.72 ± 309.56 (+27.5%) *
		O	936.21 ± 62.18 (−10.9%)
		OT	964.46 ± 35.38 (−8.2%)
	Heading-flowering	CK	1112.87 ± 25.02
		T	1412.70 ± 5.08 (+26.9%) **
		O	870.94 ± 17.08 (−21.7%) *
		OT	1008.19 ± 56.73 (−9.4%)
	Grain filling-maturity	CK	864.35 ± 6.77
		T	759.61 ± 64.72 (−12.1%) *
		O	588.22 ± 78.84 (−31.9%) **
		OT	609.01 ± 31.68 (−29.5%) **
	Whole growth stage	CK	890.70 ± 23.21
		T	1019.36 ± 85.93 (+14.4%) *
		O	704.61 ± 13.32 (−20.9%) **
		OT	758.78 ± 33.48 (−14.8%) *
Soybean	Branching	CK	1046.71 ± 105.45
		T	1123.03 ± 101.88 (+7.3%)
		O	881.31 ± 71.99 (−15.8%)
		OT	952.61 ± 78.37 (−9.0%)
	Flowering-pod	CK	4267.64 ± 307.79
		T	5305.85 ± 222.64 (+24.3%) **
		O	2740.76 ± 462.05 (−35.8%) **
		OT	342.75 ± 16.85 (−92.0%) **
	Grain filling-maturity	CK	3916.66 ± 2.00
		T	4522.57 ± 56.23 (+15.5%) **
		O	2745.45 ± 474.87 (−29.9%) **
		OT	3095.37 ± 111.01 (−21.0%) **
	Whole growth stage	CK	3077.00 ± 68.11
		T	3650.48 ± 59.00 (+18.6%) **
		O	2122.51 ± 222.35 (−31.0%) **
		OT	2399.88 ± 90.89 (−22.0%) **

Data are the mean values ± SE (percent of increase (+)/decrease (−), compared to CK). The symbol * and ** represent the significant difference between treatments and CK, at *p* < 0.05 and *p* < 0.01 levels, respectively.

**Table 3:** Effect of warming and elevated O_3_ concentration on mean CO_2_ emission fluxes from soil (mg·m^−2^·h^−1^).

Crop	Growth Stages	Treatments	The Mean CO_2_ Emission Fluxes
Wheat	Turning green	CK	221.44 ± 1.23
		T	252.77 ± 20.20 (+14.1%)
		O	171.79 ± 23.51 (−22.4%) *
		OT	206.07 ± 25.12 (−6.9%)
	Elongation-Booting	CK	286.15 ± 19.28
		T	329.79 ± 3.38 (+15.3%) **
		O	237.31±0.16 (−17.1%) **
		OT	289.53 ± 0.59 (+1.2%)
	Heading-flowering	CK	321.56 ± 28.61
		T	380.27 ± 57.63 (+18.3%) *
		O	276.23 ± 30.20 (−14.1%)
		OT	273.97 ± 5.37 (−14.8%)
	Grain filling-maturity	CK	366.49 ± 10.11
		T	387.41 ± 4.31 (+5.7%) *
		O	271.90 ± 3.48 (−25.8%) **
		OT	289.02 ± 6.95 (−21.1%) **
	Whole growth stage	CK	298.91 ± 9.14
		T	337.56 ± 19.23 (+12.9%) **
		O	239.30 ± 0.84 (−19.9%) **
		OT	264.65 ± 6.53 (−11.5%) **
Soybean	Branching	CK	365.61 ± 29.50
		T	400.23 ± 52.07 (+9.5%)
		O	289.84 ± 39.23 (−20.7%) *
		OT	347.85 ± 9.84 (−4.9%)
	Flowering-pod	CK	395.29 ± 7.85
		T	430.66 ± 39.70 (+8.9%)
		O	314.82 ± 15.13 (−20.4%) *
		OT	342.75 ± 16.85 (−13.3%) *
	Grain filling-maturity	CK	349.55 ± 36.37
		T	371.56 ± 22.94 (+6.3%)
		O	275.33 ± 38.17 (−21.2%) **
		OT	309.10 ± 0.51 (−11.6%)
	Whole growth stage	CK	370.15 ± 4.90
		T	400.82 ± 38.24 (+8.3%)
		O	293.33 ± 30.85 (−20.8%) *
		OT	333.23 ± 2.17 (−10.0%)

Data are the mean values ± SE (percent of increase (+)/decrease (−), compared to CK). The symbol * and ** represent the significant difference between treatments and CK, at *p* < 0.05 and *p* < 0.01 levels, respectively.

**Table 4:** Effect of warming and elevated O_3_ concentration on biomass of wheat and soybean (grams per base frame).

Crop	Growth Stages	Treatments	Shoot Biomass	Root Biomass	Total Biomass
Wheat	Elongation-booting	CK	12.10 ± 2.14	1.25 ± 0.13	13.35 ± 2.27
		T	18.05 ± 1.49 (+49.2%) **	1.28 ± 0.18 (+2.4%)	19.33 ± 1.32 (+44.8) **
		O	10.23 ± 2.19 (−15.5%)	1.04 ± 0.08 (−16.8%)	11.27 ± 2.25 (−15.6%)
		OT	16.56 ± 2.70 (+36.9%) *	1.02 ± 0.05 (−18.4%) *	17.58 ± 2.66 (31.7%) *
	Heading-flowering	CK	15.27 ± 1.59	1.74 ± 0.28	17.01 ± 1.74
		T	20.38 ± 1.57 (+33.5) *	1.95 ± 0.40 (+12.1%)	22.33 ± 1.97 (+31.3%) *
		O	14.80 ± 2.74 (−3.1%)	1.47 ± 0.29 (−15.5%)	16.27 ± 2.92 (−4.4%)
		OT	20.34 ± 1.58 (+33.2%) *	1.24 ± 0.58 (−28.7%)	21.58 ± 2.02 (+26.9%) *
	Grain filling	CK	24.08 ± 1.55	2.50 ± 0.43	26.58 ± 1.87
		T	27.97 ± 1.76 (+16.2%) *	3.07 ± 0.43 (+22.8%)	31.04 ± 2.06 (+16.8%) **
		O	18.62 ± 1.79 (−22.7%) **	2.05 ± 0.30 (−18.0%)	20.67 ± 1.49 (−22.2%) **
		OT	24.86 ± 2.39 (+3.2%)	2.33 ± 0.71 (−6.8%)	27.19 ± 2.15 (+2.3%)
	Maturity	CK	35.54 ± 1.89	2.47 ± 0.41	38.01 ± 1.68
		T	29.40 ± 2.36 (−17.3%) *	0.74 ± 0.18 (−70%) **	30.14 ± 2.41 (−20.7%) **
		O	27.37 ± 1.86 (−23.0%) **	1.53 ± 0.14 (−38.1%) **	28.90 ± 1.75 (−24.0%) **
		OT	26.04 ± 3.09 (−26.7%) **	0.97 ± 0.16 (−60.7) **	27.01 ± 3.21 (−28.9) **
Soybean	Maturity	CK	83.90 ± 8.28	4.13 ± 0.45	88.03 ± 8.56
		T	74.40 ± 10.20 (−11.3%)	4.07 ± 0.42 (−1.5%)	78.47 ± 10.03 (−10.9%)
		O	52.71 ± 14.10 (−37.2%) **	2.86 ± 0.64 (−30.8%)	55.57±14.69 (−36.9%)*
		OT	50.37 ± 13.29 (−40.0%) **	3.30 ± 0.60 (−20.1%)	53.67±13.89 (−39.0)**

Data are the mean values ± SE (percent of increase (+) /decrease (−), compared to CK). The symbol * and ** represent the significant difference between treatments and CK, at *p* < 0.05 and *p* < 0.01 levels, respectively.

## Supplementary Materials

The following are available online at https://www.mdpi.com/1660-4601/16/10/1755/s1, Figure S1: Diurnal variation of ozone concentration for each treatment: (a) Diurnal variation of ozone concentration in winter wheat growing season; (b) Diurnal variation of ozone concentration in soybean growing season.
