# Supplementary material for: Effect of Warming and Elevated O3 Concentration on CO2 Emissions in a Wheat-Soybean Rotation Cropland
Source: Int J Environ Res Public Health. 2019 May 17;16(10):1755. doi: 10.3390/ijerph16101755 (PMC6571970; doi:10.3390/ijerph16101755)
Supplement: Supplementary file 1 [file ijerph-16-01755-s001.pdf]

International Journal of
Environmental Research and Public Health

MDPI

Effect of Warming and Elevated O_3 Concentration on CO_2 Emission in a Wheat-Soybean Rotation Cropland

Yuanyuan Wang [1], Zhenghua Hu[1*], A.R.M. Towfiqul Islam[2*], Shutao Chen[1], Dongyao Shang[1] and Ying Xue[1]

1 Collaborative Innovation Center on Forecast and Evaluation of Meteorological Disasters, School of Applied Meteorology, Nanjing University of Information Science & Technology, Nanjing 210044, China; wangyuanyuan855@163.com (Y.W.); chenstyf@aliyun.com (S.C.); 20171203270@nuist.edu.cn (D.S.); Pamtale2741176@gmail.com (Y.X.)

2 Department of Disaster Management, Disaster Management E-Learning Centre, Begum Rokeya University, Rangpur 5400, Bangladesh; towfiq_dm@brur.ac.bd

* Correspondence: zhhu@nuist.edu.cn (Z.H.); Tel.: + 86-(0)25-58731193 (Z.H.) ; towfiq_dm@brur.ac.bd (A.R.M.T.I.)

Supplementary materials

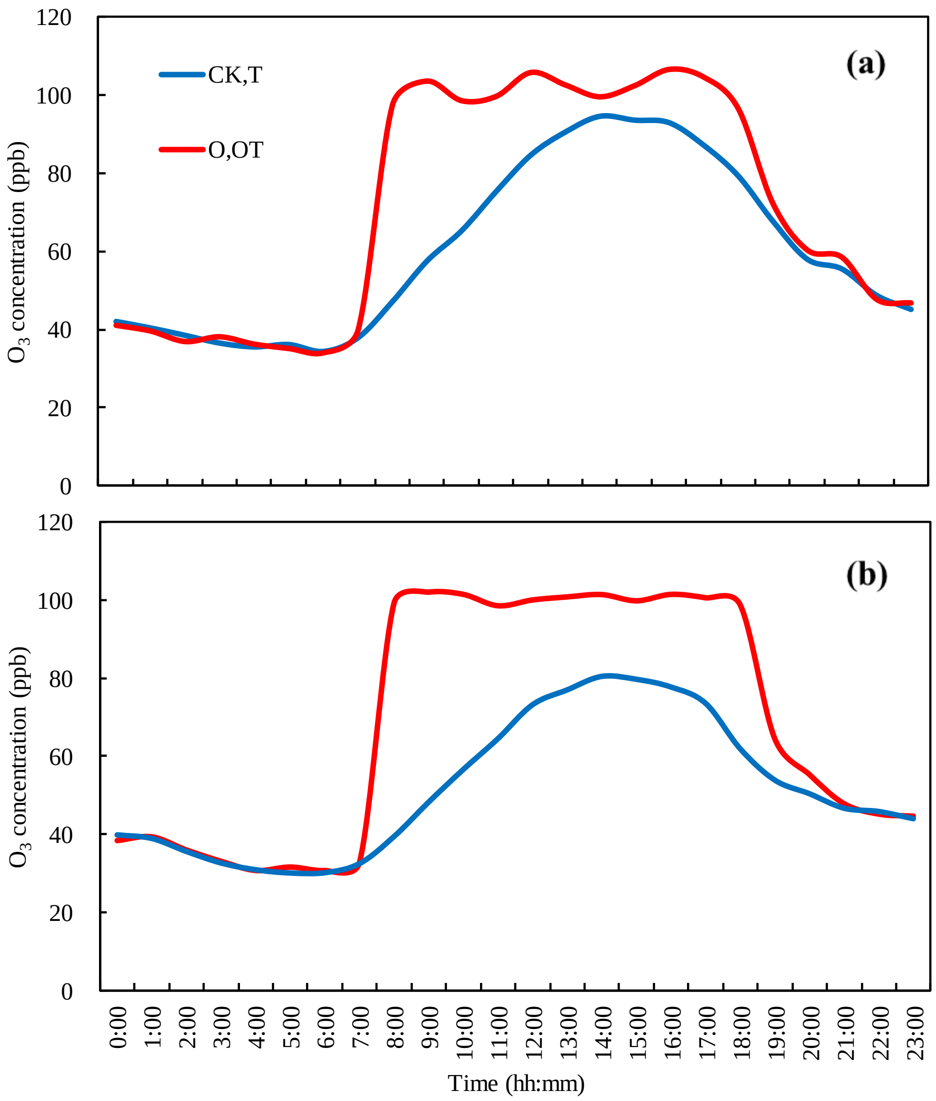

Figure 1. Diurnal variation of ozone concentration for each treatment: (a) Diurnal variation of ozone concentration in winter wheat growing season; (b) Diurnal variation of ozone concentration in soybean growing season.
